# Mechanism for Local‐Atomic Structure Changes in Chalcogenide‐based Threshold‐Switching Devices

**DOI:** 10.1002/advs.202404035

**Published:** 2024-06-20

**Authors:** Minwoo Choi, Ha‐Jun Sung, Bonwon Koo, Jong‐Bong Park, Wooyoung Yang, Youngjae Kang, Yongyoung Park, Yongnam Ham, Dong‐Jin Yun, Dongho Ahn, Kiyeon Yang, Chang Seung Lee

**Affiliations:** ^1^ Thin Film Technical Unit Samsung Advanced Institute of Technology (SAIT) Samsung Electronics Suwon‐si 16677 South Korea; ^2^ Analytical Engineering Group Samsung Advanced Institute of Technology (SAIT) Samsung Electronics Suwon‐si 16677 South Korea; ^3^ Semiconductor R&D Center Samsung Electronics Hwaseong‐si 18448 South Korea

**Keywords:** amorphous chalcogenide, defect analysis, local structure, threshold switch, trap dynamics

## Abstract

Threshold‐switching devices based on amorphous chalcogenides are considered for use as selector devices in 3D crossbar memories. However, the fundamental understanding of amorphous chalcogenide is hindered owing to the complexity of the local structures and difficulties in the trap analysis of multinary compounds. Furthermore, after threshold switching, the local structures gradually evolve to more stable energy states owing to the unstable homopolar bonds. Herein, based on trap analysis, DFT simulations, and operando XPS analysis, it is determined that the threshold switching mechanism is deeply related to the charged state of Se–Se homopolar defects. A threshold switching device is demonstrated with an excellent performance through the modification of the local structure via the addition of alloying elements and investigating the time‐dependent trap evolution. The results concerning the trap dynamics of local atomic structures in threshold switching phenomena may be used to improve the design of amorphous chalcogenides.

## Graphical abstract

This work investigates amorphous chalcogenides, focusing on local structural evolution and proposing a novel threshold switching mechanism based on Se^2−^ defects. By replacing unstable Ge─Ge and Se─Se homopolar bonds with stable S─Ge and In─Se heteropolar bonds, significant improvements in threshold‐switching devices are achieved. These findings offer insights into the design and predictive modeling of amorphous materials.

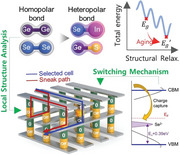

## 1 Introduction

Recently, 3D crossbar array memory has attracted attention because it has a high integration density owing to its stacking architecture and simplified two‐terminal structure, which makes it suitable for use in emerging technologies such as high‐density storage‐class memory (SCM), embedded memory, neuromorphic devices, analog computing, and artificial intelligence.^[^ ^1^, ^2^ ^]^ Notably, a selector device is necessary which can effectively reduce the sneak current. Ovonic threshold switches (OTSs) based on amorphous chalcogenide have been reported as promising selector devices owing to their unique nonlinear conductivity, high current density, fast speed (<10 ns), and large on–off ratios.^[^ ^3^, ^4^, ^5^ ^]^ However, a fundamental understanding of local atomic structures and switching mechanism of amorphous OTS has not been achieved owing to difficulties in amorphous thin film analysis and complexity in short‐range bonding configurations, which differ from the structure of the crystal materials.^[^ ^6^ ^]^ The properties of the amorphous thin film are totally changed by various trap states resulting from their local atomic structure; analysis of the local structure of amorphous material is one of the key approaches for engineering the properties of chalcogenide thin film.^[^ ^7^ ^]^ Furthermore, threshold switching under high electrical field leads to a configuration change of local structure to the energetically meta‐stable state while atomically stabilizing and recovering over time; this phenomenon is called structural relaxation.^[^ ^8^, ^9^ ^]^ The change in the bonding configuration of amorphous chalcogenides over time affects their material properties, including the band gap, resistance, and capacitance.^[^ ^10^, ^11^, ^12^ ^]^ Consequently, threshold voltage *V_th_* drift occurrs which can negatively affect crossbar array memory by reducing the read–write window margin and time‐dependent memory stability.^[^ ^13^ ^]^ Therefore, to control the properties of an amorphous chalcogenide device, it is important to understand the trap dynamics and the nature of the bonding configuration. However, few relevant studies have been conducted thus far. Hence, it is necessary to develop an analytical model of trap dynamics and local structural changes for amorphous chalcogenide materials.^[^ ^14^, ^15^ ^]^

In this study, we conducted an Ab initio molecular dynamics (AIMD) simulation and experimental analysis to investigate the origin of trap generation and alloying mechanism, thus establishing a strategy for material design. We modified the local atomic bond structures in a Ge‐As‐Se system by adding In and S atoms. We conducted simulations to predict the changes in the physical properties of the amorphous films produced using different alloying processes based on the change in the bonding configurations and trap dynamics. We then experimentally verified the local structure and bonding nature of the amorphous thin film using X‐ray adsorption fine structure (XAFS) analysis. Moreover, we calculated the trap density using temperature‐dependent current–voltage *I–V* measurements based on the thermally assisted hopping‐transport model. Furthermore, optical pumping measurement was utilized for trap profile analysis enabled by the wide wavelength laser scanning range, and operando X‐ray photoelectron spectroscopy (XPS) analysis was conducted to investigate the threshold‐switching mechanism. Finally, we verified the device performance via electrical measurements using the optimal alloying composition ratio.

## 2 Results

**Figure 1:**
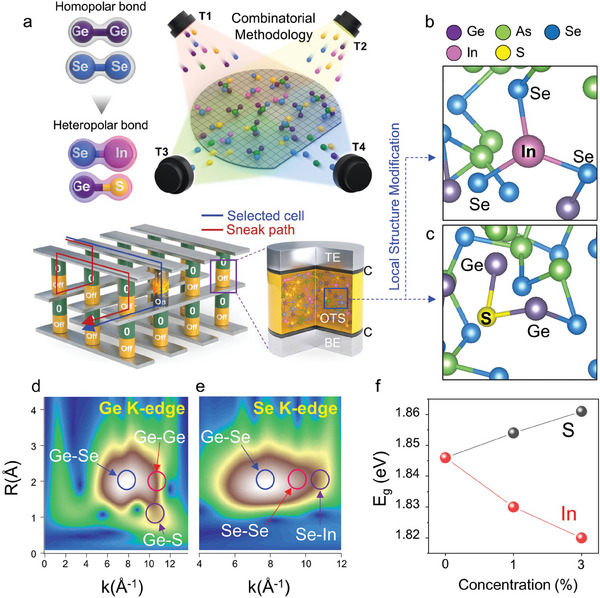
Local atomic‐structure engineering of the selector material. Schematic illustrations showing a) the deposition of the amorphous chalcogenide material using a combinatorial method and the 3D cross‐point memory (the inset shows the structure of the selector device). b,c) Molecular dynamics simulation results showing the local atomic structure of the Ge‐As‐Se‐In‐S amorphous system with In‐Se and S‐Ge heteropolar bonds. d,e) WT‐XAFS of the Ge‐As‐Se‐In‐S film. S formed heteropolar bonds with Ge, which changed the local bonding configuration at the Ge K‐edge. Similarly, In formed heteropolar bonds with Se, which changed the local bonds at the Se K‐edge. f) Optical bandgap properties of chalcogenide materials according to atomic concentrations.

A sputtering technique based on a combinatorial method was used to deposit the multicomponent chalcogenide materials. This approach allowed high throughput thin film screening by designing a compositional library array on a wafer. The deposition process and the cell structure of crossbar device composed of selector and memory elements (1S 1R) is illustrated in **Figure** **1**a. Selectors are required to prevent sneak currents with low off current (*I_off_*) for low power consumption. In addition, low *V_th_* drift, high endurance and high thermal stability are necessary for reliable device.^[^ ^16^, ^17^ ^]^ The inset in Figure 1a shows the structure of a simple two‐terminal selector device. Figure S1a (Supporting Information) shows the experimentally validated results of change in *I_off_* and *V_th_* drift for pre‐screening of alloying atoms. Generally, the addition of metallic atoms tends to improve *V_th_* drift with *I_off_* degradation. Among the alloying atoms, it could be observed that Indium (In) significantly improves *V_th_* drift while suppressing *I_off_* simultaneously. For the purpose of *I_off_* improvement, alloying atoms with the potential to decrease *I_off_* were predicted through the technology computer‐aided design (TCAD) and density functional theory (DFT) calculations as shown in Figure S1b (Supporting Information). Owing to the trap‐limited conduction mechanism of the threshold switching device, *I_off_* primarily depends on the trap density.^[^ ^18^, ^19^ ^]^ Especially, Sulfur (S) is calculated to have a significantly improved *I_off_* with compatible alloying controllability. Therefore, we considered In and S as promising alloying elements for the Ge‐As‐Se thin film system. We generate the amorphous model for Ge‐As‐Se‐In‐S through melt‐and‐quench AIMD to predict the time‐dependent short‐range reconfiguration of local structure in the amorphous material, as shown in Figure S2 (Supporting Information). In complex quinary amorphous systems, homopolar and heteropolar bonding are randomly distributed, and these local bond structures significantly affect to properties of the amorphous materials.^[^ ^20^ ^]^ The AIMD simulations revealed that each additional atom tended to form different heteropolar bonds, as shown in Figure 1b,c. The In atoms bonded preferentially with Se atoms and the bonds were characterized by threefold symmetry. By contrast, the S atoms bonded preferentially with the Ge atoms, and the bonds were characterized by twofold symmetry. In the amorphous Ge‐As‐Se system, the formation of unstable homopolar bonds caused gradual changes in the local structure over time. Therefore, a fundamental analysis of the homopolar bonding behavior and trap dynamics was required to understand the properties of amorphous materials.^[^ ^21^ ^]^

Figure 1d,e and S3 (Supporting Information) show the changes in the local structure of the Ge‐As‐Se‐In‐S thin film, which we analyzed using wavelet transform extended X‐ray absorption fine structure (WT‐EXAFS).^[^ ^22^ ^]^ The wavelet transform method greatly increased the resolution of the bonding characterization compared to that of conventional 1D EXAFS. The analysis of the Ge‐K edge showed that the Ge─Ge homopolar bonds were replaced by S─Ge heteropolar bonds after S addition. Similarly, the analysis of the Se K‐edge showed that the Se─Se homopolar bonds were replaced by In─Se heteropolar bonds after In addition. These results were consistent with the simulation, which showed that Ge tended to form local bonds with S, whereas Se tended to form local bonds with In, rather than forming unstable Ge─Ge or Se─Se homopolar bonds, respectively. The XPS analysis shown in Figure S4 (Supporting Information) revealed that S formed heteropolar bonds with metal atoms via the p orbitals. To clearly observe the S 2p peak near the Se 3p main peak, we analyzed the local bonding configuration by adding a relatively high concentration of 10% S. This result also indicates the possibility of S─Ge bonding formation as well. Figure 1f shows that the optical bandgap *E_g_* of the amorphous chalcogenides varied as a function of the S‐ and In‐atomic concentrations. Notably, *E_g_* increased as the S‐atomic concentration increased and decreased as the In‐atomic concentration increased. In general, the bandgap of an amorphous material is associated with the trap states near the mobility edge, which are called tail states. When the tail states near the valence band maximum (VBM) and conduction band minimum (CBM) increase, the bandgap tends to decrease. By contrast, when the tail states decrease, the bandgap tends to increase.^[^ ^23^ ^]^ Thus, the controlling properties of amorphous chalcogenide are possible through changes of trap states such as bandgap modulation utilized to control the *I_off_* of selector devices.

**Figure 2:**
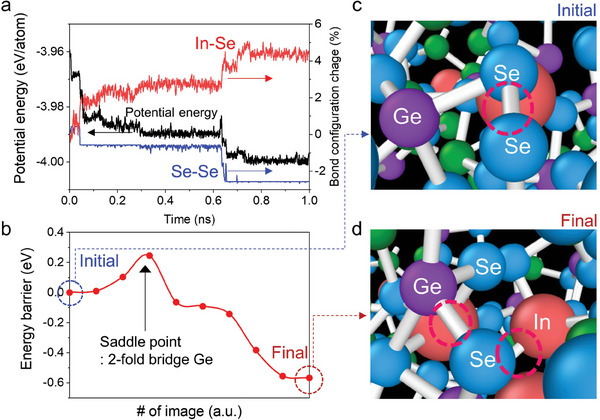
Trap dynamics analysis. a) Change in bonding configuration from Se─Se homopolar bonding to In─Se heteropolar bonding as a function of time. The change of bonding configuration can be determined by comparing the number of chemical bonds at each MD steps with that of initial structure. The black line denotes the potential energy which is the total energy of the amorphous structure without kinetic energy. b) NEB barrier for the stabilization of amorphous chalcogenide through Se─Se homopolar annihilation near 0.62 ns region. Images of the molecular dynamics simulations for the c) initial and d) final states matched with the NEB barrier plot. The Se─Se homopolar bonds were replaced by Ge─Se and In─Se heteropolar bonds.

We examine the reconfiguration of short‐range order structure by time evolution. The results of the AIMD of Ge‐As‐Se‐In are shown in **Figure** **2**a. Over a few nanoseconds, the number of Se─Se homopolar bonds decreased and the number of In─Se bonds increased as the total potential energy of the amorphous system stabilized. Typically, amorphous materials experience a phenomenon known as structural relaxation, where the energy of the local atomic structures tends to stabilize over time. The results showed that the Se─Se homopolar bonds increased the instability of the amorphous materials. Therefore, the stability of the amorphous material was improved by reducing the number of Se─Se homopolar bonds by minimizing the defects resulting from metastable local atomic structures. Indium atoms changed the Se─Se homopolar bonds to In─Se heteropolar bonds, which effectively suppressed structural relaxation and drift phenomena. Figure 2b shows the nudged elastic band (NEB) barrier during structural relaxation.^[^ ^24^ ^]^ The saddle points were located at the twofold bridge Ge within the Se─Se homopolar bond and the energy barrier was calculated to be 0.24 eV per atom. As shown by the AIMD simulation, unstable Se─Se homopolar bonds were present in the initial state. However, in the final state, the Se─Se bonds were broken and new heteropolar bonds, such as Ge─Se and In─Se, were formed, as shown in Figure 2c,d. Therefore, the amount of homopolar bonding should be reduced to improve the structural stability of amorphous chalcogenides. This indicates that the properties of chalcogenide glass can be modulated by controlling the local bonds in the amorphous structure, which paves the way for a diverse range of applications in trap‐based electronics.

As shown in Figure S5a–d (Supporting Information), we calculated the projected density of states (PDOS) profiles by applying DFT for various atomic ratios of S and In. Each concentration for the S/In ratio used in the calculation is indicated in Figure S5 (Supporting Information). When S/In = 2, the tail states near the VBM decreased, which increased the bandgap. Therefore, the *I_off_* of the selector device decreased, as predicted by the trap‐limited conduction mechanism.^[^ ^25^ ^]^ Conversely, when S/In > 2, additional S‐related defect states were generated, which increased *I_off_*. The simulation results for the changes in the bonding configuration as a function of S/In are shown in Figure S5e (Supporting Information). Notably, when S/In > 2, the heteropolar and homopolar bonding configurations remained relatively constant. Therefore, S/In = 2 should be used to prevent degradation of *I_off_* effectively reducing homopolar bonding to maintain the time‐dependent stability of the amorphous structure. To get ideal electronic properties in Ge‐As‐Se‐In‐S OTS, not only the ratio of S/In but also the overall content of S and In are important. When In concentration is fixed to ≈1.5%, S/In = 2 is found to be optimal ratio to improve the *I_off_* (Figure S6, Supporting Information). However, the *I_off_* at S of 3% and In of 1.5% is 0.86 nA which is much larger than 0.29 nA at S of 1% and In of 0.75%. We think the optimum value of S/In ratio can be changed by In concentration. At In 0.75%, even when 1% of S is added (S/In = 1.33), it sufficiently exhibits the effect of reducing *I_off_*. Finally, we used a high‐throughput combinatorial screening methodology to examine the S/In ratio in Ge‐As‐Se‐In‐S OTS by mapping thin film libraries deposited on a wafer. Furthermore, we used X‐ray diffraction (XRD) peak analysis and Raman spectroscopy to determine the crystalline temperature *T_x_* and glass transition temperature *T_g_* of the film, which were >500 and 385 °C, respectively, as shown in Figure S7 (Supporting Information). These values offer thermal budget of the deposition and integration processes of crossbar memory.

**Figure 3:**
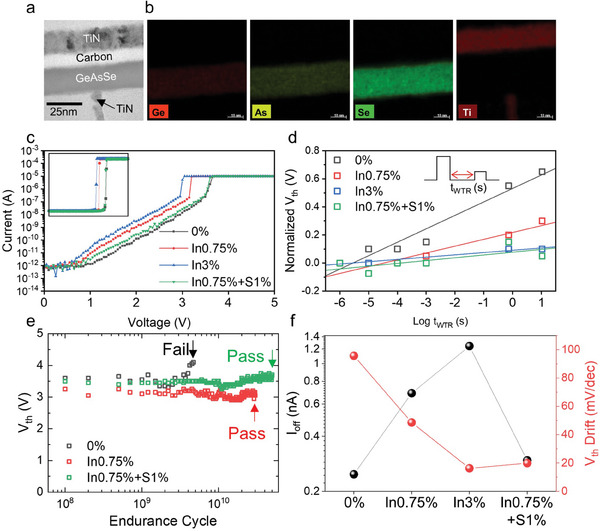
Electrical characterization of the Ge‐As‐Se‐In‐S selector device. a) TEM analysis of the cross‐section and b) EDS mapping of the selector device. Plots of the c) *I–V* characteristics, d) *V*_th_ drift, e) endurance, and f) *I_off_* and *V_th_* drift trend with alloying elements, measured at elevated temperature of 85 °C. The *I*_off_ and *V*_th_ drift exhibit trade‐off characteristics. However, both can be improved simultaneously with In and S addition.

We fabricated a selector device to evaluate the properties of the optimized thin films. **Figure** **3**a shows a transmission electron microscopy (TEM) image of the cross‐section of the T‐shaped selector device. The Ge‐As‐Se active layer was sandwiched between the top and bottom TiN electrodes, and a carbon layer served as the diffusion barrier. Energy‐dispersive X‐ray spectroscopy (EDS) mapping revealed that the main components (Ge, As, and Se) were homogeneously distributed across the thin film, as shown in Figure 3b. Figure S8 (Supporting Information) summarizes the methods used to characterize the four main parameters of the selector: *V_th_* and *I_off_* were evaluated via direct current *I–V* measurements and the *V_th_* drift and cyclic endurance were evaluated using time‐dependent pulsed *I*–*V* measurements. The *I–V* characteristics of the threshold‐switching device are shown in Figure 3c. As the In‐atomic concentration increased, *V_th_* decreased and the subthreshold current increased. Furthermore, if we add In and S together, *V_th_* and the subthreshold current returned to values similar to those of the pristine state, which contributed to improving *I_off_*. Notably, the reduction in the tail states near the VBM caused by the addition of S effectively improves *I_off_*. The *V_th_* drift characteristics are shown in Figure 3d. As the In‐ and S‐atomic concentrations increased, the slope of *V–t* curve decreased as a function of writing to read time (t_WTR_), which indicates that the *V_th_* drift was improved. This phenomenon occurred owing to the formation of heteropolar In─Se and S─Ge bonds, which resulted from the reduction in the number of unstable Se─Se and Ge─Ge homopolar bonds. In addition, the endurance cycle property is shown in Figure 3e. The incorporation of stable heteropolar bonding, such as In─Se and S─Ge, is expected to elevate the energy barrier for atomic migration.^[^ ^26^ ^]^ Consequently, alloying In and S enhanced the endurance cycle property. Figure 3f shows the trends of device properties associated with addition of In and S atoms. Generally, *I_off_* is defined as current under 50% of *V_th_* condition due to the half‐bias array operation of crossbar memory. However, in this research, we evaluated the *I_off_* value under 60% of *V_th_* condition for ensuring a margin for device performance in practical memory operations. The Ge‐As‐Se ternary alloy exhibits the lowest *I_off_* values without the addition of any alloying elements. However, surprisingly, alloying with a combination of In and S results in comparable characteristics in terms of *I_off_*, along with an enhanced *V_th_* drift property. Alloying atoms such as In helps to stabilize Se‐related bonding, and improve *V_th_* drift but exhibit a trade‐off characteristic by increasing trap state and degrading *I_off_*. Interestingly, combining S with In suppresses trap generation, improving *I_off_* and simultaneously enhancing *V_th_* drift through the stabilization of Ge─Ge and Se─Se homopolar bonds. Therefore, the Ge‐As‐Se‐In‐S device exhibited excellent properties including a low *I_off_* (0.29 nA), extremely low *V_th_* drift (19.5 mV dec^−1^), and high endurance (>50 G) even measuring at an elevated temperature of 85 °C.

We also used contour mapping using combinatorial methodology to optimize the electrical properties according to the Ge‐As‐Se concentration ratio, as shown in Figure S9 (Supporting Information). The *I_off_* and *V_th_* drift exhibited a trade‐off relationship. The value of *I_off_* was optimized when the Ge concentration was high (Ge > 25%), whereas the *V_th_* drift was optimized when the Ge concentration was low (Ge < 15%). Therefore, based on the mapping results, an optimal Ge‐As‐Se ratio was proposed for the Ge‐As‐Se‐In‐S system, which had a low *I_off_* (0.27 nA), low *V_th_* drift (17.4 mV dec^−1^), and high endurance (>70 G), as shown in Table S1 (Supporting Information). Figure S10 (Supporting Information) displays the previous state‐of‐the‐art of the T‐shape structured selector device.^[^ ^27^, ^28^, ^29^, ^30^, ^31^, ^32^ ^]^ When compared to the results from the previous researches, it can be observed that the Ge‐As‐Se‐In‐S selector device exhibits significantly low *I_off_* characteristics and superior endurance cycle properties.

**Figure 4:**
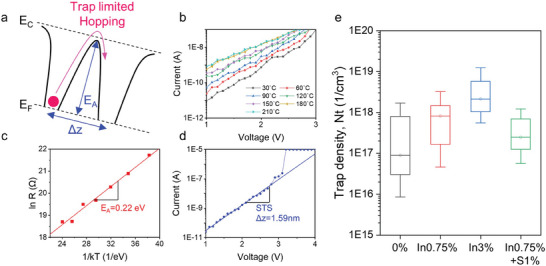
Trap density analysis using the trap‐limited conduction model. a) Illustration of the hopping conduction mechanism in the amorphous chalcogenide material. b) Temperature‐dependent *I–V* characteristics. c) Activation energy calculation form Arrhenius plot. d) STS calculation from the *I–V* curve. e) Calculation of *N_t_* using the trap limited conduction model. (Parameters of *A *= 320 nm^2^, *d *= 16 nm, *T *= 300 K, *τ_0_* = 10^−11^ was used).

In amorphous chalcogenide selectors, electron carriers are transported through the various defect states that result from the incorrect bonding of the amorphous phase. In particular, carriers are transported to different trap states via the hopping mechanism, which is assisted by the electrical field and thermal energy in the subthreshold region, as shown in **Figure** **4**a.^[^ ^33^, ^34^ ^]^ This mechanism is called the trap‐limited conduction model and can be expressed as

(1) $$I\;=\;2qAN_{t}\frac{\Delta z}{\tau_{0}}\exp\left(-\frac{E_{C}-E_{F}}{kT}\right)\sinh\left(\frac{qV_{A}}{kT}\frac{\Delta z}{2d}\right)$$

where *q* is the charge; *A* is the area of the device; *N_t_* is the trap density; *Δz* is the inter‐trap distance; *τ_0_* is the escape time for a trapped electron; *E_C_* and *E_V_* are the boundary energies of the conduction and valance bands, respectively; *k* is the Boltzmann constant; *T* is the temperature; *V_A_* is the applied voltage; and *d* is the film thickness. Because amorphous chalcogenide materials adhere to the thermally assisted conduction model, the activation energy *E_A_* can be determined using the temperature‐dependent *I–V* measurements and expressed by the Arrhenius equation (Figure 4b,c). That is,

(2) $$\ln R\;=\;\ln R_{0}+E_{A}\frac{1}{kT}$$

where *R* is the resistance and *R_0_* is the pre‐exponential factor. Moreover, the subthreshold slope (STS) is given by

(3) $$\mathrm{STS}\;=\frac{\partial logI}{\partial V}\;=\frac{q}{kT}\;\frac{\Delta z}{2d}$$

which can be used to calculate Δ*z* for the conduction model in Equation 1 (Figure 4d). By substituting each calculated parameter into Equation 1, we can determine *N_t_*.

Figure 4e shows the calculated trap density for the Ge‐As‐Se‐In‐S selector device. As the In concentration increased, *N_t_* gradually increased. This phenomenon can be attributed to the formation of In‐related trap states, which reduced the bandgap and increased *I_off_*. By contrast, as the S concentration increased, *N_t_* decreased, as shown by the analysis of the bonding nature and the reduction in the shallow trap state near the VBM. These observations indicate that the trap density of amorphous chalcogenides can be controlled by altering the local atomic structure, which affects the device characteristics.

**Figure 5:**
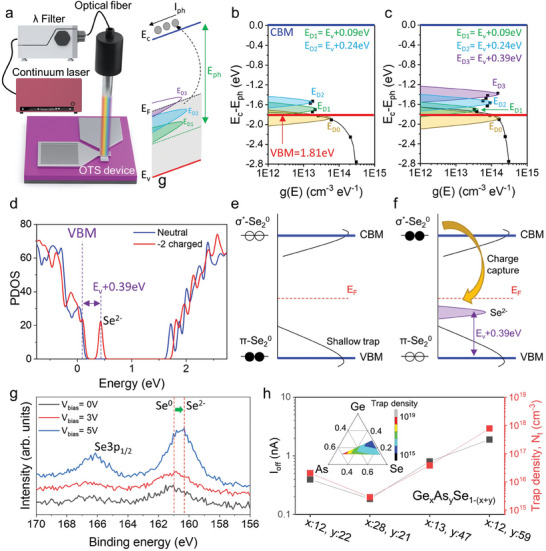
Trap profile analysis via optical pumping measurements. a) Schematic of the aligned laser source and electrical measurement system. Extracted DOS profile b) before and c) after the FF. d) PDOS of the neutral and 2‐ charged state after FF, determined via the DFT calculation. Illustration of the band structure of threshold switching materials e) before and f) after the FF. The Se^2−^ defects were produced by the deep trap state, where the energy level matched the optical pumping measurement. g) Operando XPS spectra of Se 3p with in situ applied voltage bias in Ge‐As‐Se thin film. h) Plot of correlation between *I*_off_ and *N*_t_ for different Ge‐As‐Se ratios. Inset image indicate the contour maps for *N_t_*.

Previously, amorphous chalcogenide materials have been difficult to analyze quantitatively because an analytical methodology has not been established for irregular trap distributions. Therefore, we introduced a new approach and measured the trap profile by determining the density of states (DOS) via photonic *I–V* measurements (**Figure** **5**a; Figure S11, Supporting Information). The photocurrent was measured to determine the number of excited photo‐carriers using the drift current model.^[^ ^35^, ^36^, ^37^ ^]^ Subsequently, the trap profile was calculated using the chain rule of energy mapping and the photo‐carrier equations

(4) $$n_{ph}=\frac{I_{ph}d}{q\mu VA}\;$$

and

(5) $$g\;\left(E\right)=\frac{dn_{ph}}{dE_{ph}}\;=\frac{dn_{ph}}{d\lambda }\;\frac{d\lambda }{dE_{ph}}$$

Furthermore, the trap density (*N_t_*) from optical pumping measurement was evaluated using the following equation

(6) $$N_{t}=\int_{E_{V}}^{E_{C}-E_{ph}}g\left(E\right)dE\;$$

where *n_ph_*, *I_ph_*, *E_ph_*, and *µ* denote the number of photo carriers, photo‐current, photon energy, and carrier mobility, respectively. Figure 5b,c show the trap profiles of the threshold‐switching device before and after the first firing (FF), which were measured using the optical pumping method. The VBM was defined by the equation *E_C_* – *E_V_* = 1.81 eV, which is the value of the optical bandgap measured via ellipsometry. Before the FF, the trap profile showed a tail state, denoted *E_D0_*, and two peaks, denoted *E_D1_* and *E_D2_*, were observed in the in‐gap state region. After the FF, the *E_D0_*, *E_D1_*, and *E_D2_* trap states were activated and *N_t_* increased. In particular, additional E_D3_ defects formed at the deep trap level (*E_V_* + 0.39 eV). These observations are consistent with the results of the DFT calculations, as shown in Figure 5d. Interestingly, the energy level of the Se‐related defect was matched to that of the optical pumping measurement, that is, *E_V_* + 0.39 eV notated as *E_D3_* defects. Figure 5e,f illustrate the electronic structure schematically before and after the FF respectively. The Se─Se homopolar can capture electrons and becomes the negatively charged defect under a high‐voltage electric field. When the anti‐bonding state (σ^*^‐Se_2_^0^) of the Se─Se homopolar was occupied, weakening the bond. As the Se─Se bond is broken, the defect state can be understood as the p‐orbital state of an isolated Se atom. The filled anti‐bonding state is lowered and turns to a nonbonding defect level in −2 charged, near the valence band edge, notated as Se^−2^ state. Here, we note that E_D3_ defects can be well assigned to Se^−2^ nonbonding state. To experimentally validate the Se‐related defect state, operando XPS spectra of Se 3p analysis is conducted in a Ge‐As‐Se thin film under in situ applied voltage bias, as plotted in Figure 5g. Under a high applied field, it was observed that Se^0^ peak of pristine material was shifted to the Se^2−^ state. On the other hand, Ge^0^ and As^0^ peak shows no discernible displacement at the Ge 3d and As 3d respectively, even under high bias. This observation provides insight that Se‐related defects are closely related to the threshold‐switching mechanism (Figure S12, Supporting Information). This XPS analysis can be explained by DFT simulation that Se^2−^ deep traps were generated by Se─Se bond breaking after first firing. As shown in Figure S13 (Supporting Information), the total energy increased as the traps were activated following the FF. During the FF, the amorphous selector material is activated from the pristine to the excited state. In the excited state, it is energetically metastable and a significant number of trap states are generated. Over time, the metastable amorphous material experiences structural relaxation, which results in gradual stabilization accompanied by a time‐dependent drift in the material properties. Hence, we experimentally validated the threshold switching mechanism of the amorphous chalcogenide selector device from the perspective of charged defect changes. Furthermore, the *I_off_* and *N_t_* showed high similarity with the Ge‐As‐Se composition ratio, as shown in Figure 5h. The trap density, *N_t_*, was calculated from the trap profile obtained through optical pumping measurement by integrating *g(E)* from *E_V_* to *E_C_−E_ph_*, as shown in Equation 6. These results demonstrated that the bonding configuration significantly affected the electrical properties of the selector device.

## 3 Discussion

In this work, we studied the amorphous chalcogenide based on a deep understanding of the local structural evolution to establish the prediction model and material design. In addition, we proposed for the first time a threshold switching mechanism based on the Se^2−^ defects, which was validated theoretically and experimentally in Se‐based OTS. These findings would provide the basic information to understand the memory working mechanism of self‐selecting memory. Based on the trap dynamics analysis, we found that structural relaxation in amorphous materials is associated with instability of homopolar bonding.

Therefore, we proposed that In could replace instable Se─Se homopolar bonds with stable In─Se Heteropolar bonds, resulting in 75% improvement of V_th_ drift compared to the pristine Ge‐As‐Se system. Moreover, S could suppress the number of shallow traps and widen the bandgap. Hence, we achieved a 50% improvement in I_off_ compared to that of the Ge‐As‐Se‐In system. The relationship between the trap density and I_off_ was determined via optical pumping measurements, which showed that the S─Ge bond may be closely related to N_t_. Generally, the addition of atoms increases the trap state of chalcogenide materials. Interestingly, however, S tends to preferentially form S─Ge heteropolar bonds, suppressing trap generation. In conclusion, homopolar bond related traps have a significant effect on the electrical properties of amorphous chalcogenides. Therefore, atomic‐structure engineering based on fundamental analysis can provide insight for amorphous material design and is expected to be a key knowledge to build a simulation model for amorphous material properties prediction.

## 4 Experimental Section

### Combinatorial Methodology for Amorphous Chalcogenide Thin Films

A unique combinatorial sputtering system (CANON‐Anelva) was used for high‐throughput material deposition. This system allows 41 different compositional splits of thin‐film materials to be deposited on a single wafer. The composition of the multicomponent chalcogenides was controlled by co‐sputtering with four tilted sputtering guns. Inductively coupled plasma atomic emission spectroscopy (ICP‐AES) and X‐ray fluorescence spectrometry (thin) were used to verify the chemical composition of the co‐sputtered thin film. Moreover, a 10 nm‐thick carbon thin film was used as a capping layer to prevent oxidation and diffusion.

### Calculation Method and AIMD

Molecular dynamics simulations were performed using the Perdew–Burke–Ernzerhof (PBE) exchange‐correlation functional, as implemented in the Vienna Ab initio Simulation Package (VASP) code.^[^ ^38^ ^]^ To get more reliable band gap and defect levels, we employ the hybrid functional of Heyd–Scuseria–Ernzerhof (HSE06) for the exchange‐correlation potential.^[^ ^39^, ^40^ ^]^ In a supercell containing 100 atoms, the wave functions were expanded in plane waves up to a cutoff value of 400 eV. Gamma centered *k*‐point mesh of sampling was used for the Brillouin zone integration. The bonding configuration trajectories were examined by AIMD simulations up to 1 ns at 600 K. The migration barriers were calculated using NEB method using PBE functional. The utilization of high‐throughput screening methods can mitigate the experimental resource and accelerate the development of materials.^[^ ^41^, ^42^ ^]^ To pre‐screen the alloying elements for *I_off_* improvement, we extract the *I_off_* and *V_th_* through combined DFT and TCAD simulation with trap‐limited PF conduction and impact ionization threshold switching model. We generate 10 different amorphous structures in DFT simulation to obtain the averaged band gap and trap density without stochastic effect. Using these DFT parameters, the leakage current *I_off_* is determined as the current at the 0.6 *V_th_* in our TCAD simulations. We screened the alloying elements X fixed to 5% in Ge‐As‐Se composition and there are 64 candidates for X elements. The *ΔI_off_* is estimated by comparing the *I_off_* values of X‐Ge‐As‐Se with that of pure Ge‐As‐Se. The detailed calculation results are presented in Table S2 (Supporting Information).

### Electrical Characterization of the Selector Device

We measured the *I–V* characteristics of the selector using a two‐terminal *I–V* sweep and the source measurement unit of the Keysight 4070 parametric test system was used to evaluate *I_off_* and *V_th_*. A pulse measurement system was used to evaluate the *V_th_* drift and endurance. A Keysight 81150A was used as the pulse generator and the source for the input voltage. A Keysight CX3320A waveform analyzer was used to monitor the output current.

### DOS Extraction from the Photonic *I–V* Measurement

A supercontinuum laser (NKT Photonics, FIANIUM) with wavelengths of 440–980 nm was used as the optical pumping source. To minimize intensity variation effect, the power was controlled based on pre‐measured intensity information for each wavelength range. The laser was precisely aligned using a positioning system to ensure sufficient photon energy absorption in the device's active area. Through comparison of the differences between the current and dark current resulting from laser irradiation at various wavelengths, only the photo‐current values generated by photocarriers were specifically identified. The number of photocarriers was calculated using the drift current equation based on the measured photocurrent for each laser wavelength. Subsequently, the trap energy levels corresponding to each laser source were mapped with the change in the number of excited photocarriers as a function of the wavelength. The DOS was determined by matching the trap density and energy states.

## Conflict of Interest

The authors declare no conflict of interest.

## Author Contributions

M.C. and H.‐J.S. contributed equally to this work. C.S.L. and K.Y. planned and supervised the study. M.C. conducted most of the device characterizations, defect analyses, and manuscript writing. H‐.J.S. supported the DFT calculations and material modeling. B.K., J.B.P., W.Y., Y.K., Y.P., Y.H., and D.‐J.Y. supported the combinatorial sputter deposition, TEM, XRD, XRF, device fabrication, ICP‐AES, and XAFS analysis, respectively. D.A. supported the planning of material screening. All of the authors contributed to the data analysis.

## Supporting information

Supporting Information

## Data Availability

The data that support the findings of this study are available from the corresponding author upon reasonable request.
