# Supplementary material for: Mechanism for Local‐Atomic Structure Changes in Chalcogenide‐based Threshold‐Switching Devices
Source: Adv Sci (Weinh). 2024 Jun 20;11(32):2404035. doi: 10.1002/advs.202404035 (PMC11348050; doi:10.1002/advs.202404035)
Supplement: Supplementary file 1 — Supporting Information [file ADVS-11-2404035-s001.docx]

**Supplementary Information**

Fig. S1. Pre-screening of Alloying atoms for amorphous chalcogenide material.

Fig. S2. Molecular dynamics simulation.

Fig. S3. Analysis of the bonding nature in the Ge-As-Se-In-S system.

Fig. S4. XPS measurements of the Ge-As-Se-S amorphous films.

Fig. S5. Trap profile and bonding configuration analysis for different values of S/In.

Fig. S6. *I_off_* with S/In ratio, measured at elevated temperature of 85°C.

Fig. S7. Thermal stability of the Ge-As-Se-In-S OTS material.

Fig. S8. Electrical characterization of the selector properties.

Fig. S9. Contour maps of the electrical characteristics.

Fig. S10. Comparison of *I_off_* and endurance cycle properties of T-shape selector devices with those reported in previous state-of-the-art studies.

Fig. S11. Photonic I–V based trap profile measurement.

Fig. S12. Operando XPS measurement.

Fig. S13. Energy states of the material.

Table S1. Electrical properties of the Ge-As-Se-In-S-based selector devices.

Table S2. For the alloying elements X in Ge-As-Se composition, we perform the DFT simulation to obtain the band gap (*E_g_*) and trap density (*N_T_*) parameters as averaged values of 10 different amorphous structure.


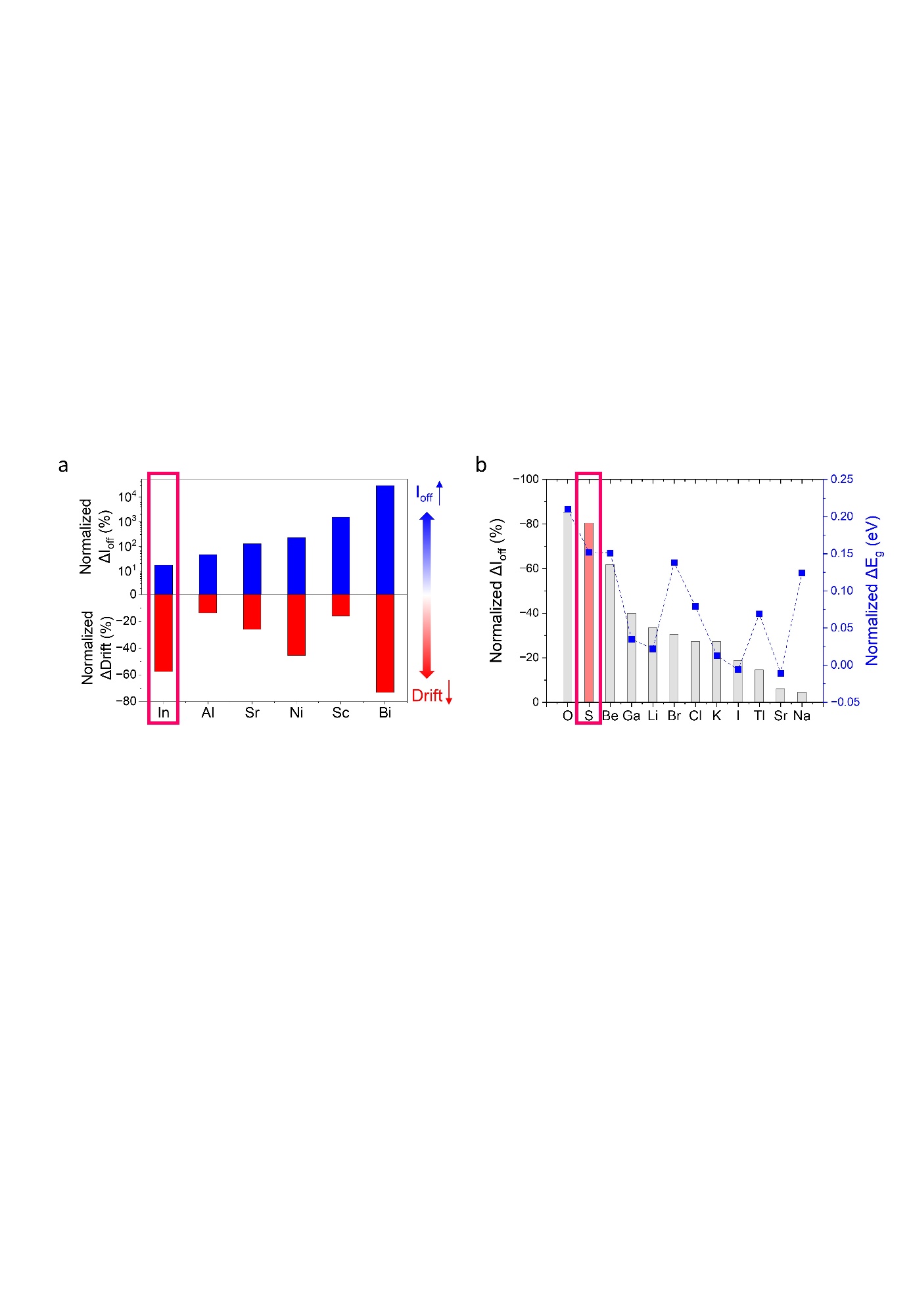


**Fig. S1.** Pre-screening of Alloying atoms for amorphous chalcogenide material. **(a)** Experimental results of change in *I_off_* and *V_th_* drift depending on alloying atoms. **(b)** TCAD and DFT simulation results for screening alloying atoms for improving *I_off_* in Ge-As-Se system.


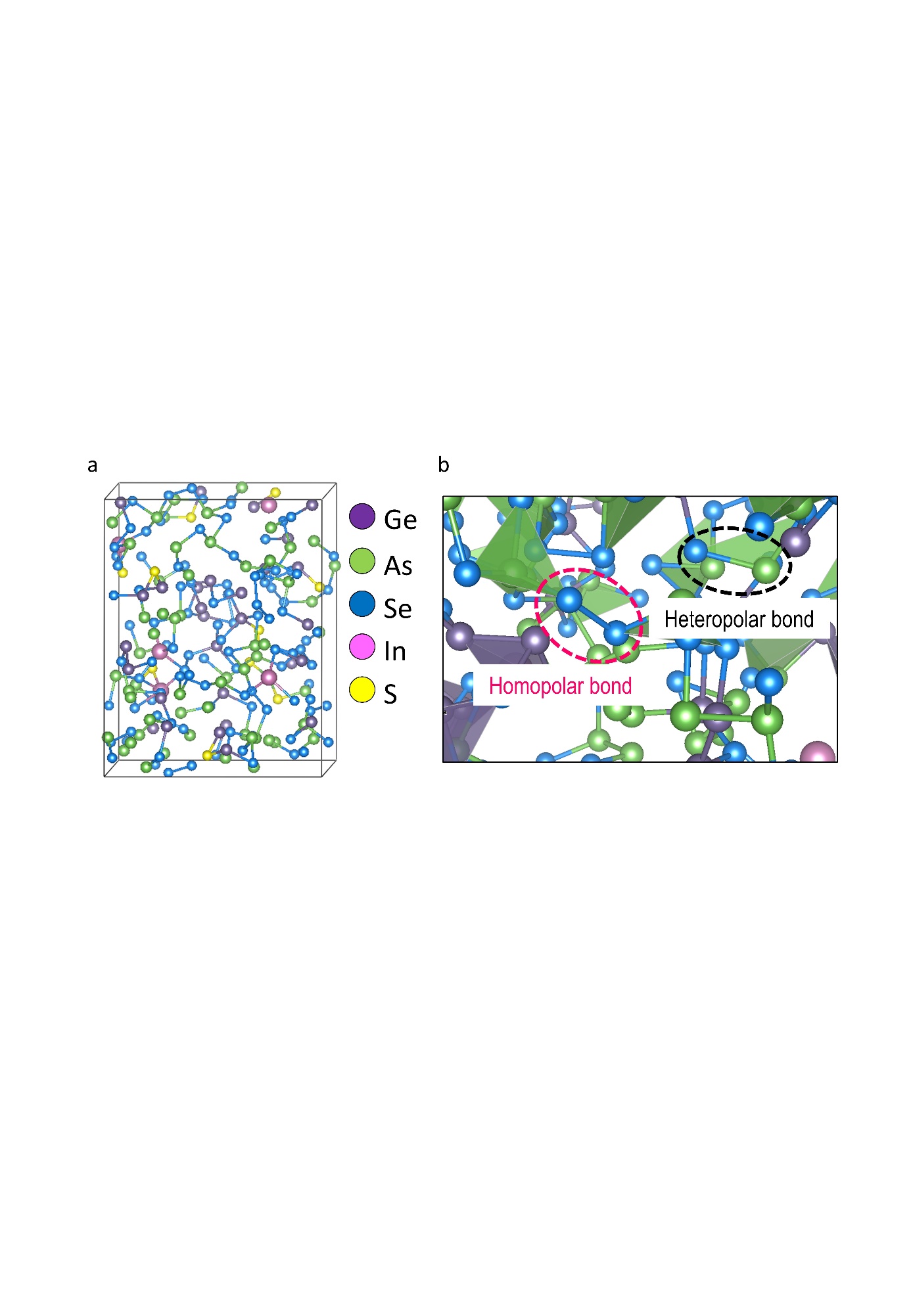


**Fig. S2.** Molecular dynamics simulation. **(a)** AIMD simulation of an amorphous Ge-As-Se-In-S system composed of 100 atoms. **(b)** Bonding configuration including homopolar and heteropolar bonds.


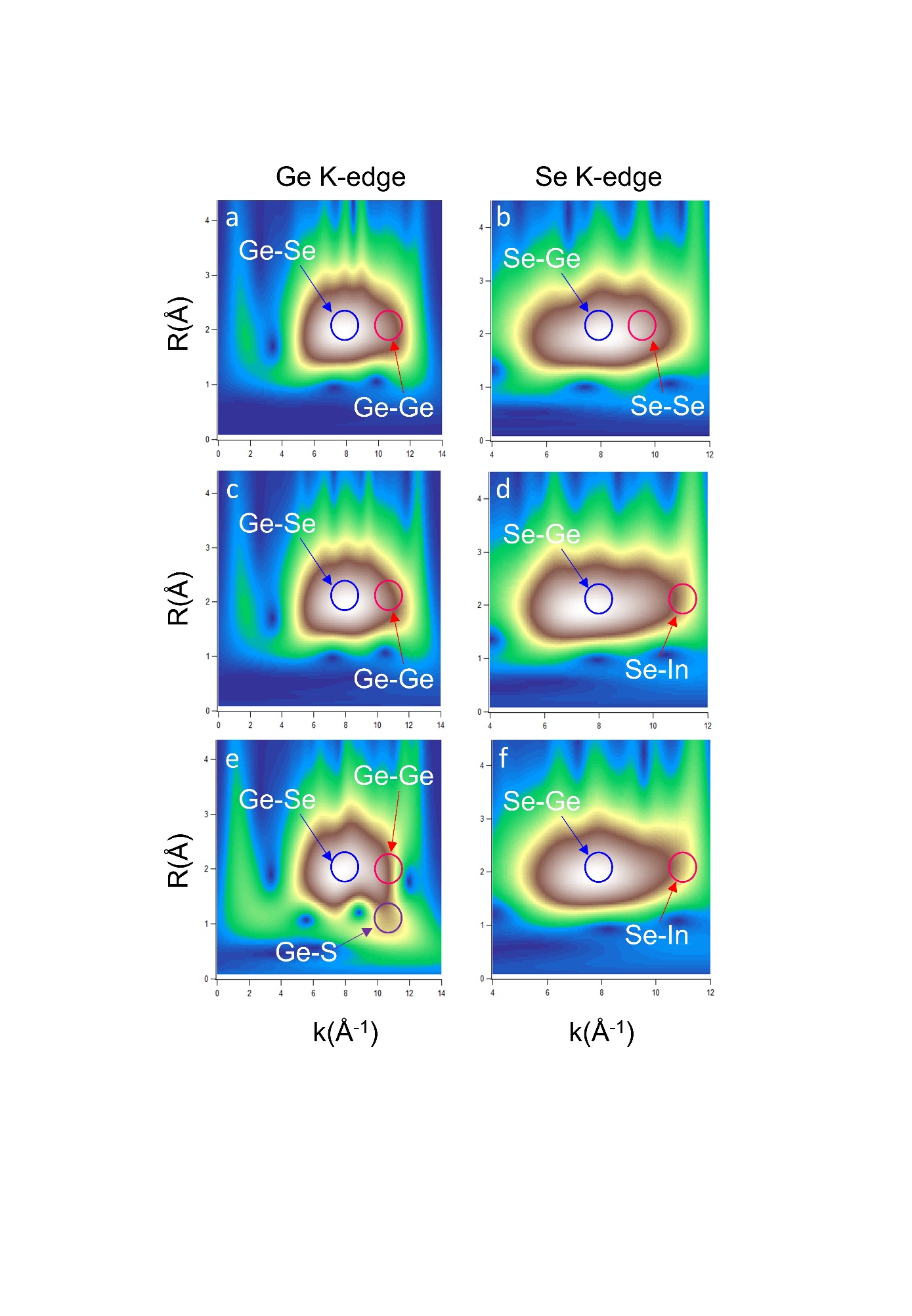


**Fig. S3.** Analysis of the bonding nature in the Ge-As-Se-In-S system. WT-XAFS of **(a, b)** Ge-As-Se, **(c, d)** Ge-As-Se-In, and **(e, f)** Ge-As-Se-In-S. S and Ge atoms combined to form S-Ge heteropolar bonds, which mainly affected the Ge K-edge. Similarly, the In and Se atoms combined to form In-Se heteropolar bonds, which mainly affected the Se K-edge


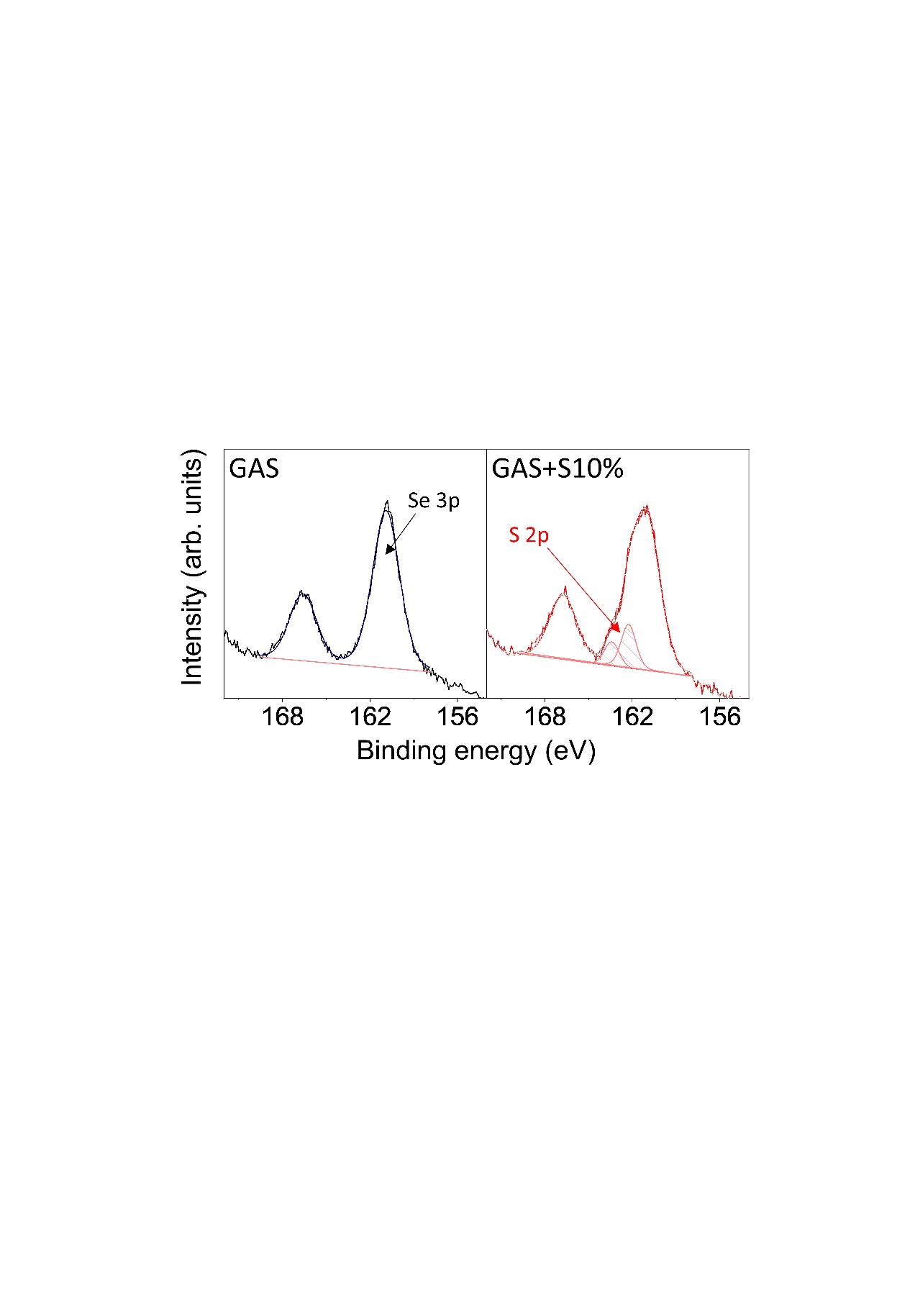


**Fig. S4.** XPS measurements of the Ge-As-Se-S amorphous films. 2p orbital peak is observed after adding 10% of S-atomic concentration.


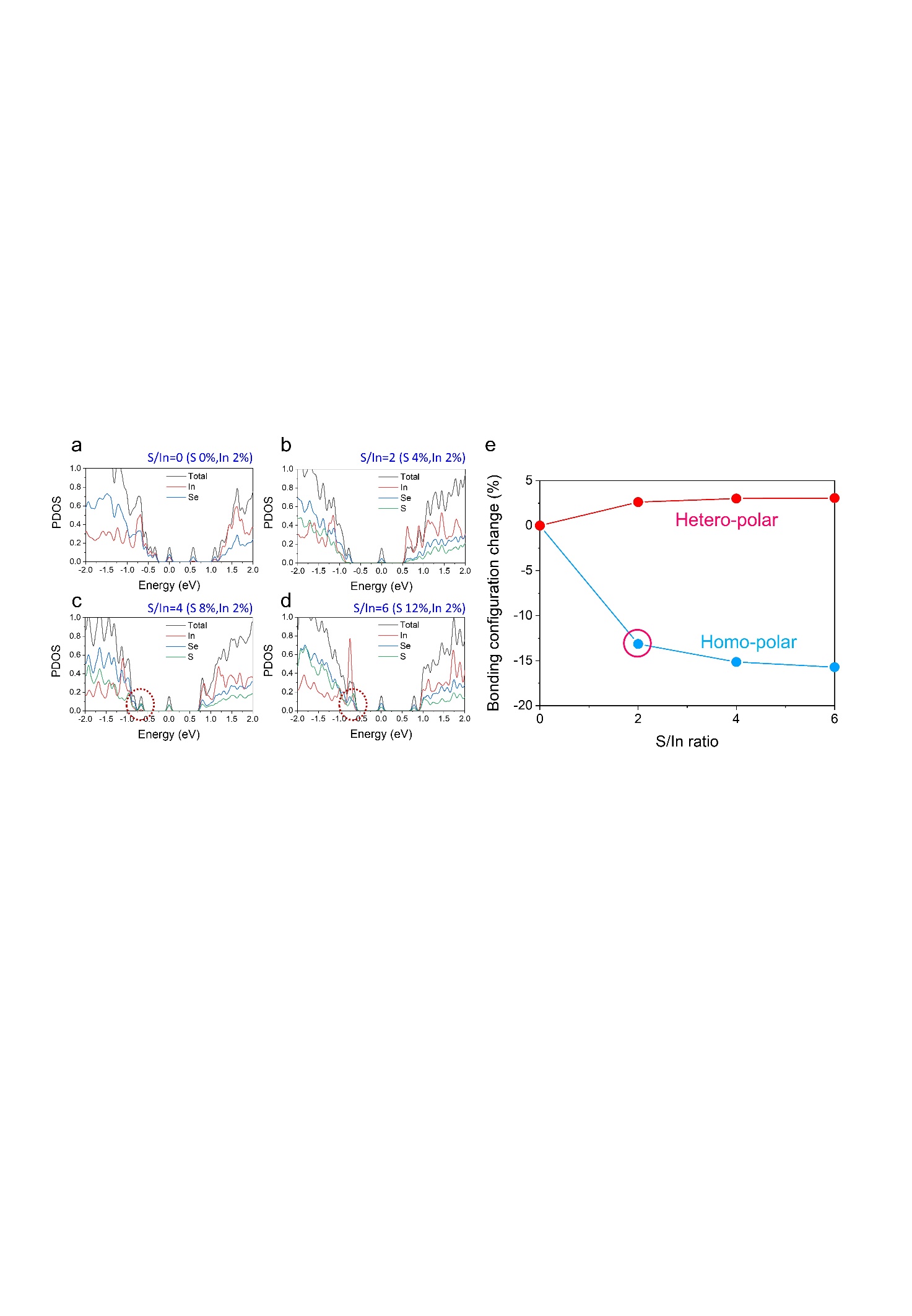


**Fig. S5.** Trap profile and bonding configuration analysis for different values of S/In. **(a–d)** Plots showing the PDOS determined from the DFT calculations. **(e)** Heteropolar–homopolar bonding configurations as a function of S/In.


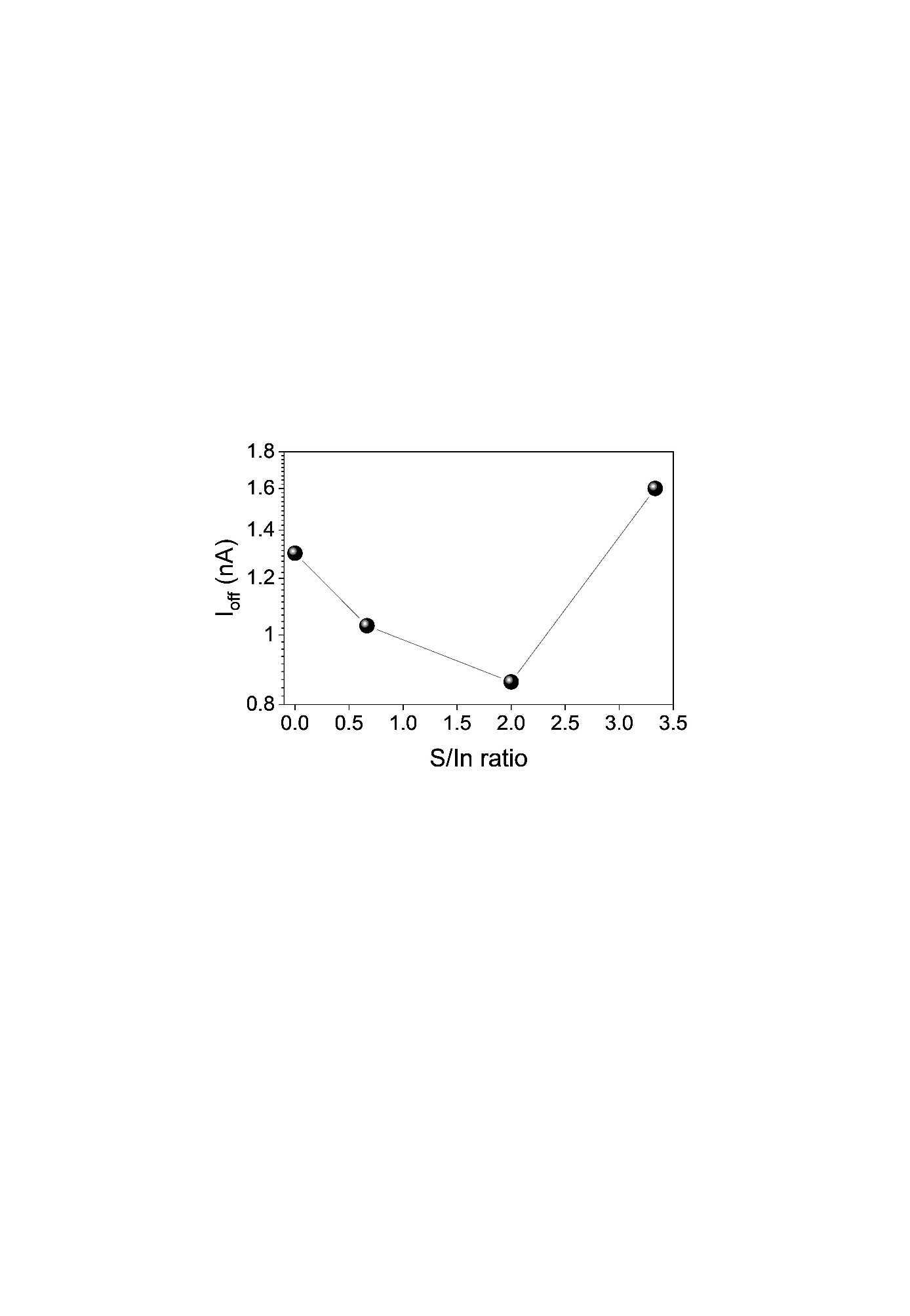


**Fig. S6.** *I_off_* with S/In ratio, measured at elevated temperature of 85°C. The In concentration was fixed at 1.5%, while the S concentration was adjusted to 0%, 1%, 3%, and 5%.


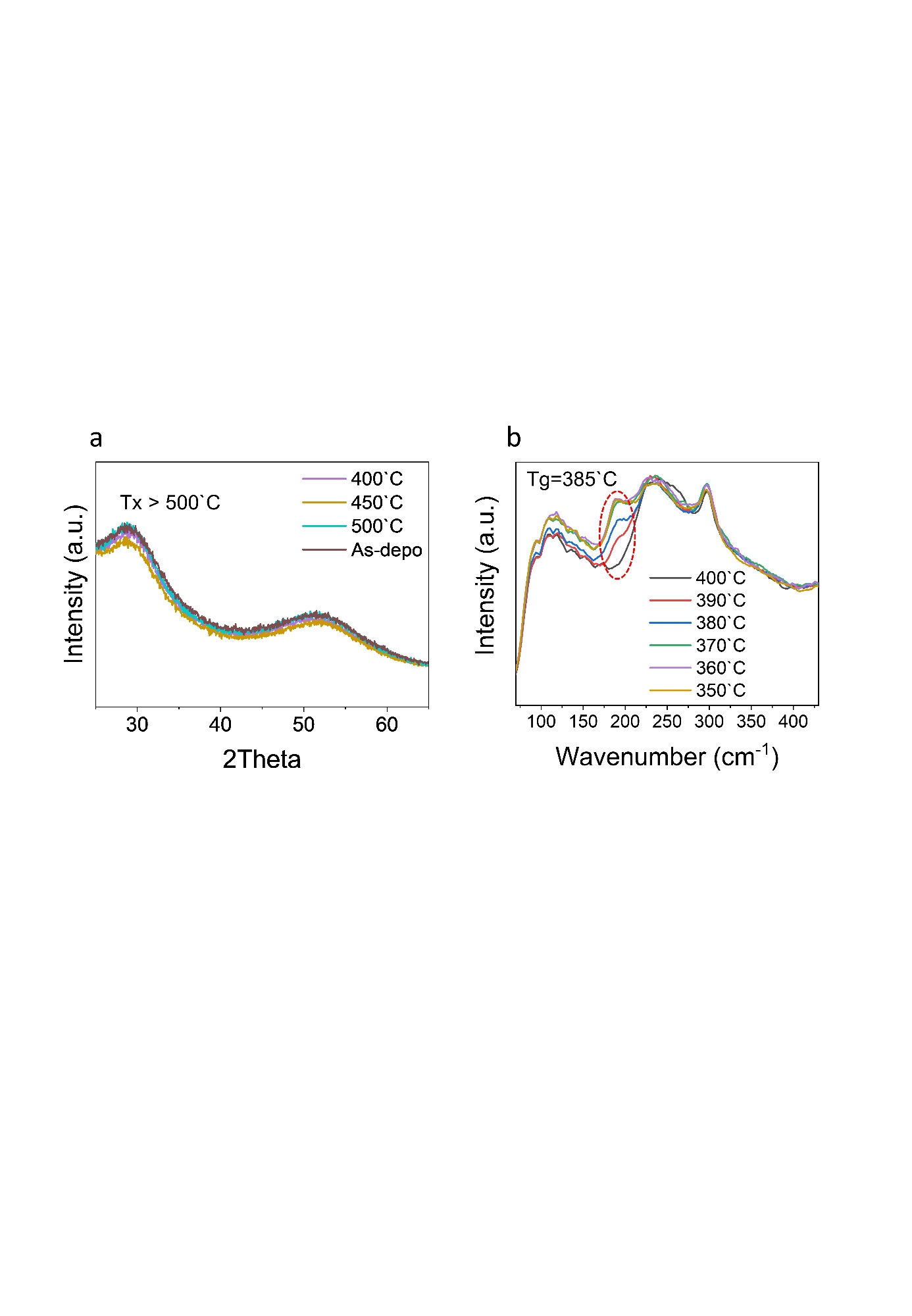


**Fig. S7.** Thermal stability of the Ge-As-Se-In-S OTS material. **(a)** Crystal temperature *T_x_* measured via XRD peak analysis and **(b)** transition glass temperature *T_g_* determined via Raman spectroscopy.


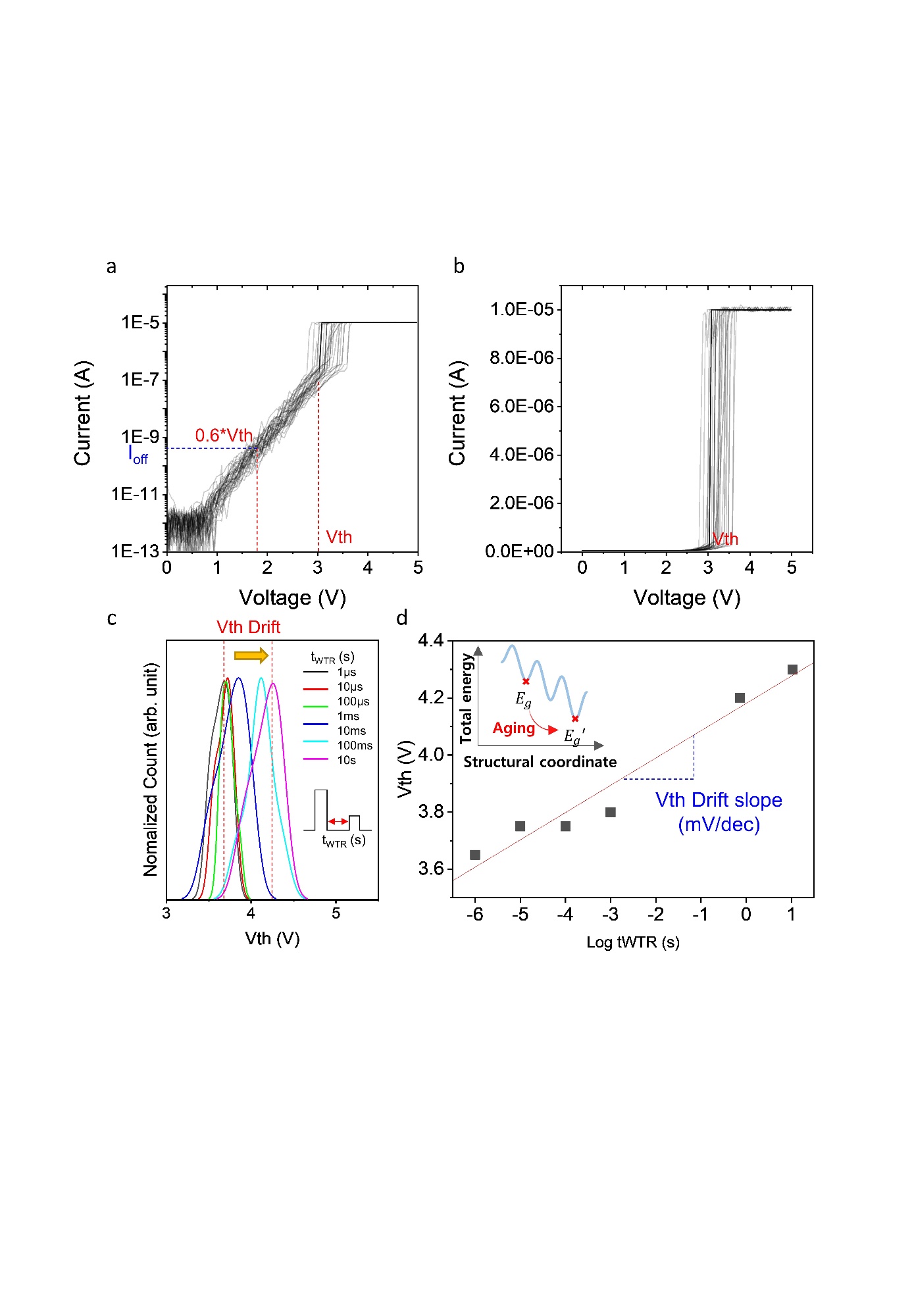


**Fig. S8.** Electrical characterization of the selector properties. Calculation of **(a, b)** *I_off_* and *V_th_* from the DC measurements and **(c, d)** *V_th_* drift from the RF measurements.


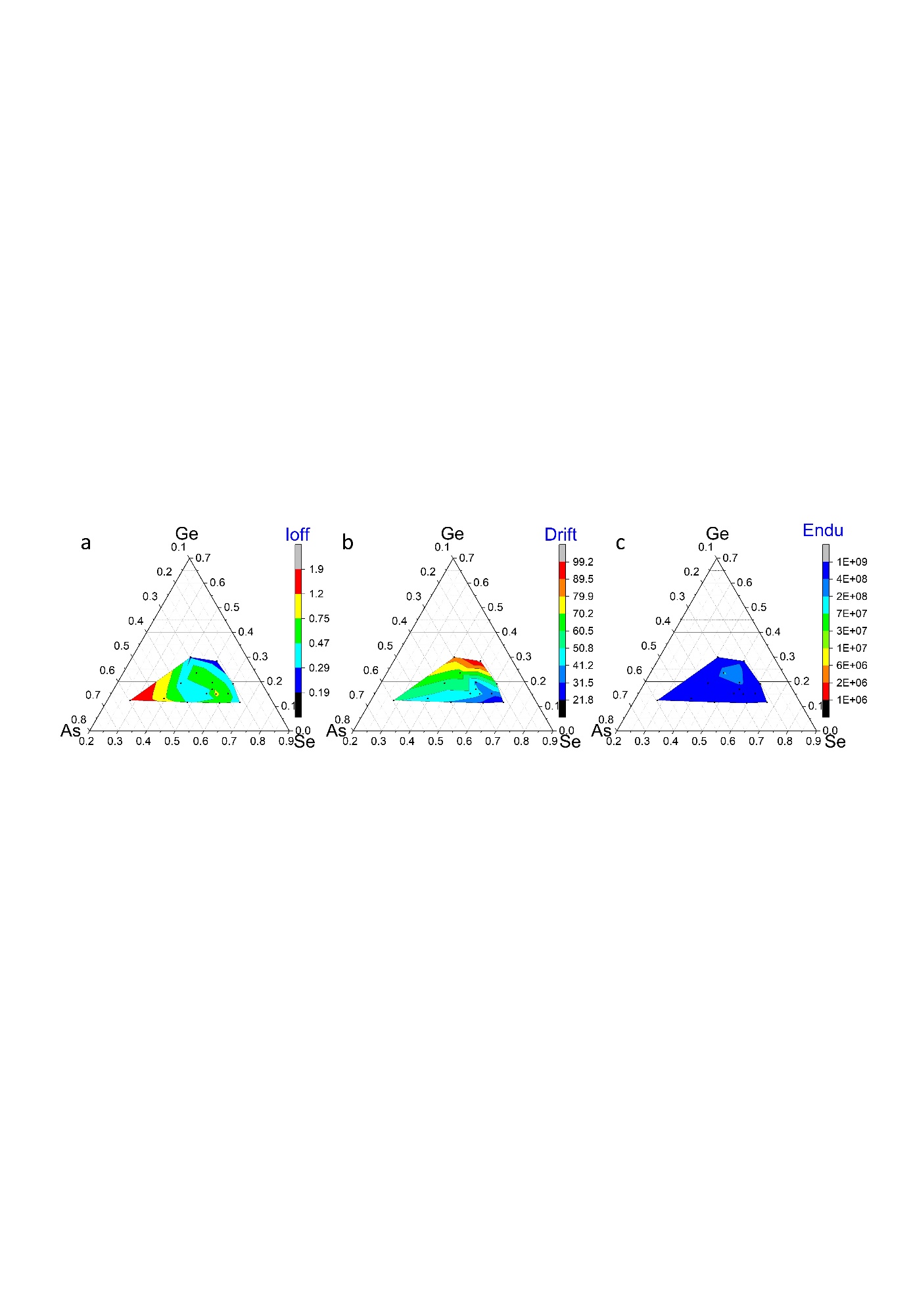


**Fig. S9.** Contour maps of the electrical characteristics. The maps for **(a)** *I_off_*, **(b)** *V_th_* drift, and **(c)** Endurance were evaluated as a function of the Ge-As-Se ratio.


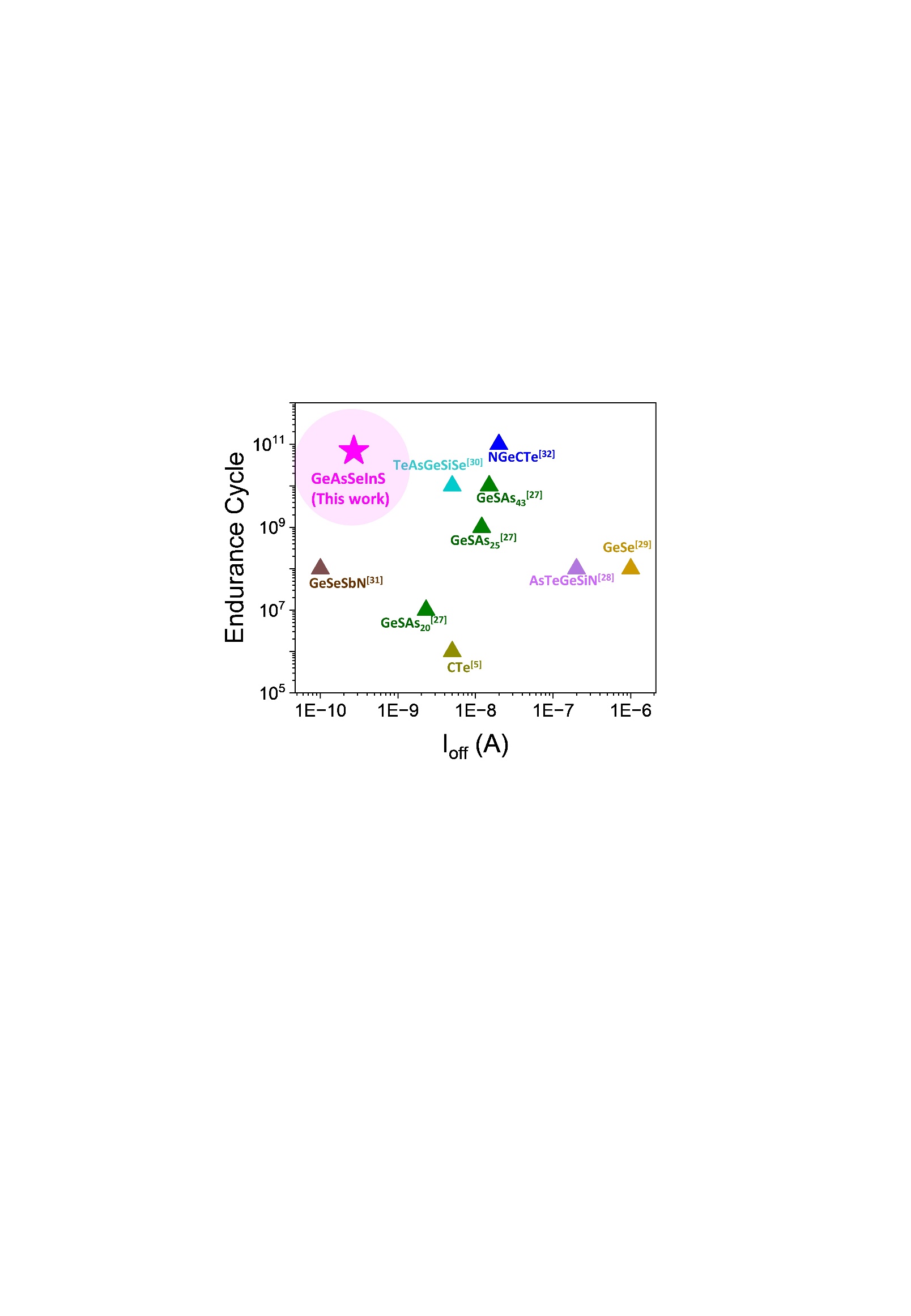


**Fig. S10.** Comparison of *I_off_* and endurance cycle properties of T-shape selector devices with those reported in previous state-of-the-art studies. The GeAsSeInS device demonstrates extremely low leakage characteristics as well as highly stable endurance during repeated operations.


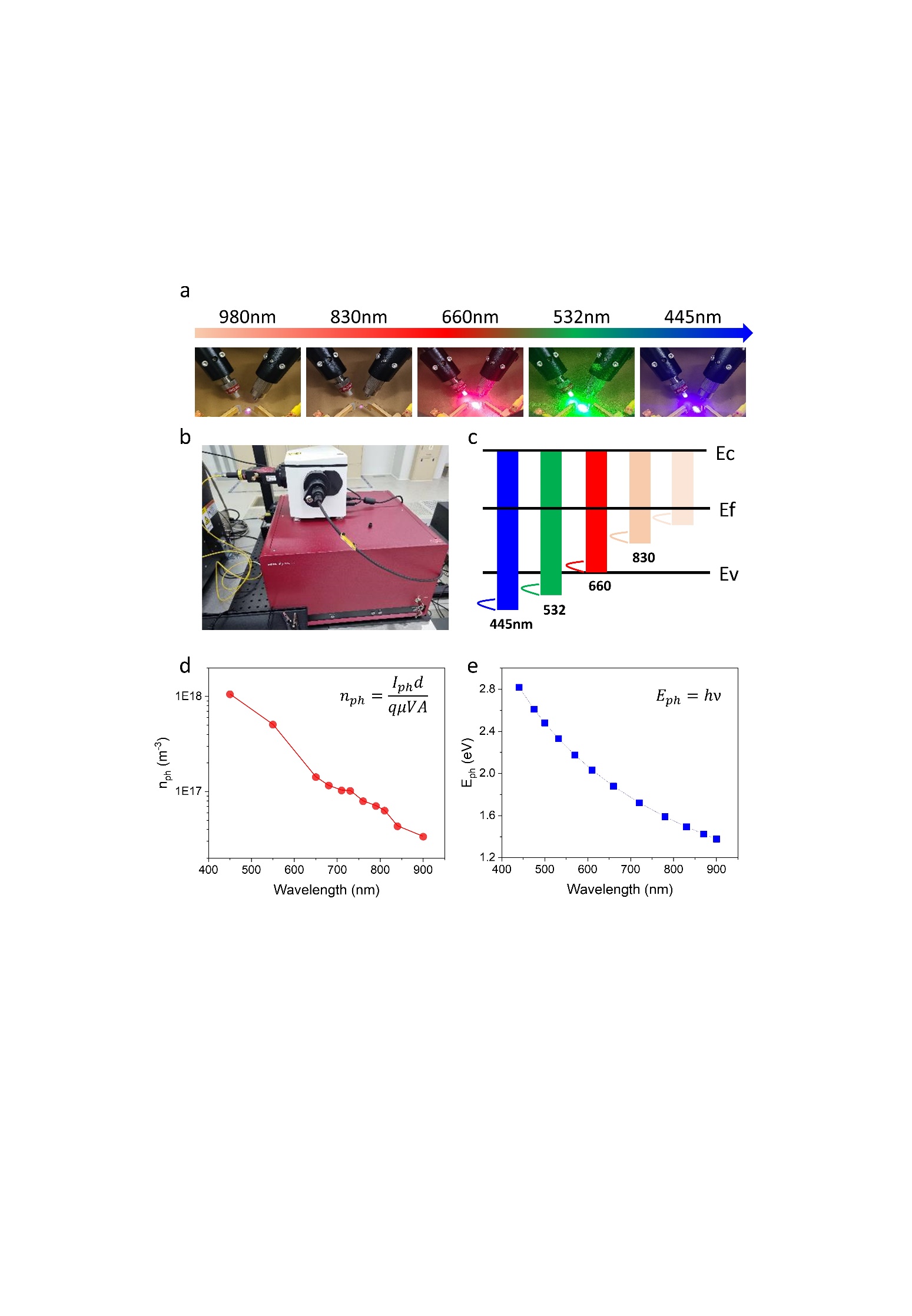


**Fig. S11.** Photonic *I–V* based trap profile measurements. Photographs of the **(a)** laser scanning at different wavelengths and **(b)** the supercontinuum laser system. **(c)** Schematic of the laser scanning method. Plots of the **(d)** number of photocarrier and **(e)** photon energy against the wavelength.


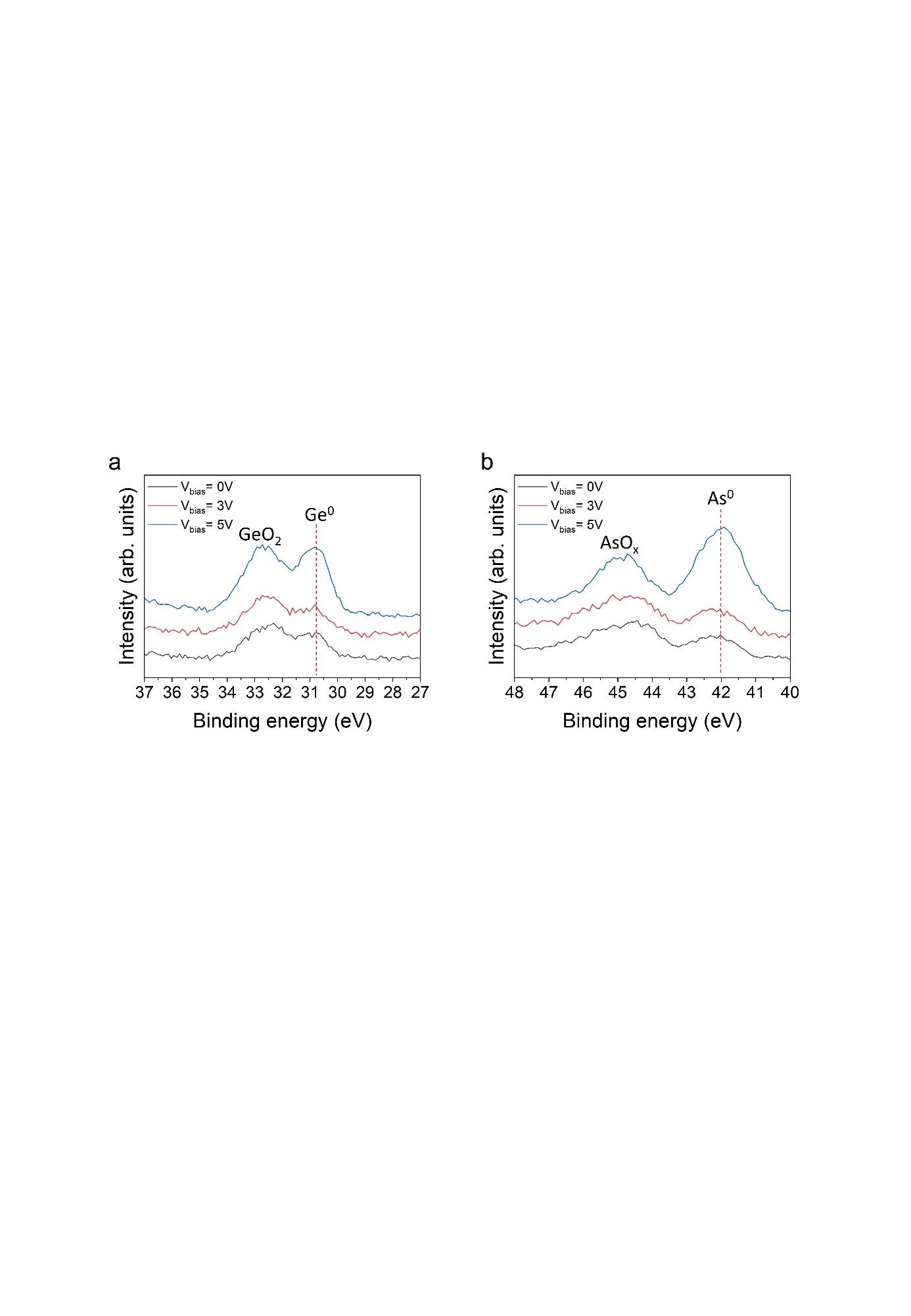


**Fig. S12.** Operando XPS measurements. XPS spectra of **(a)** Ge 3d and **(b)** As 3d in the Ge-As-Se thin film under in-situ applied voltage bias.


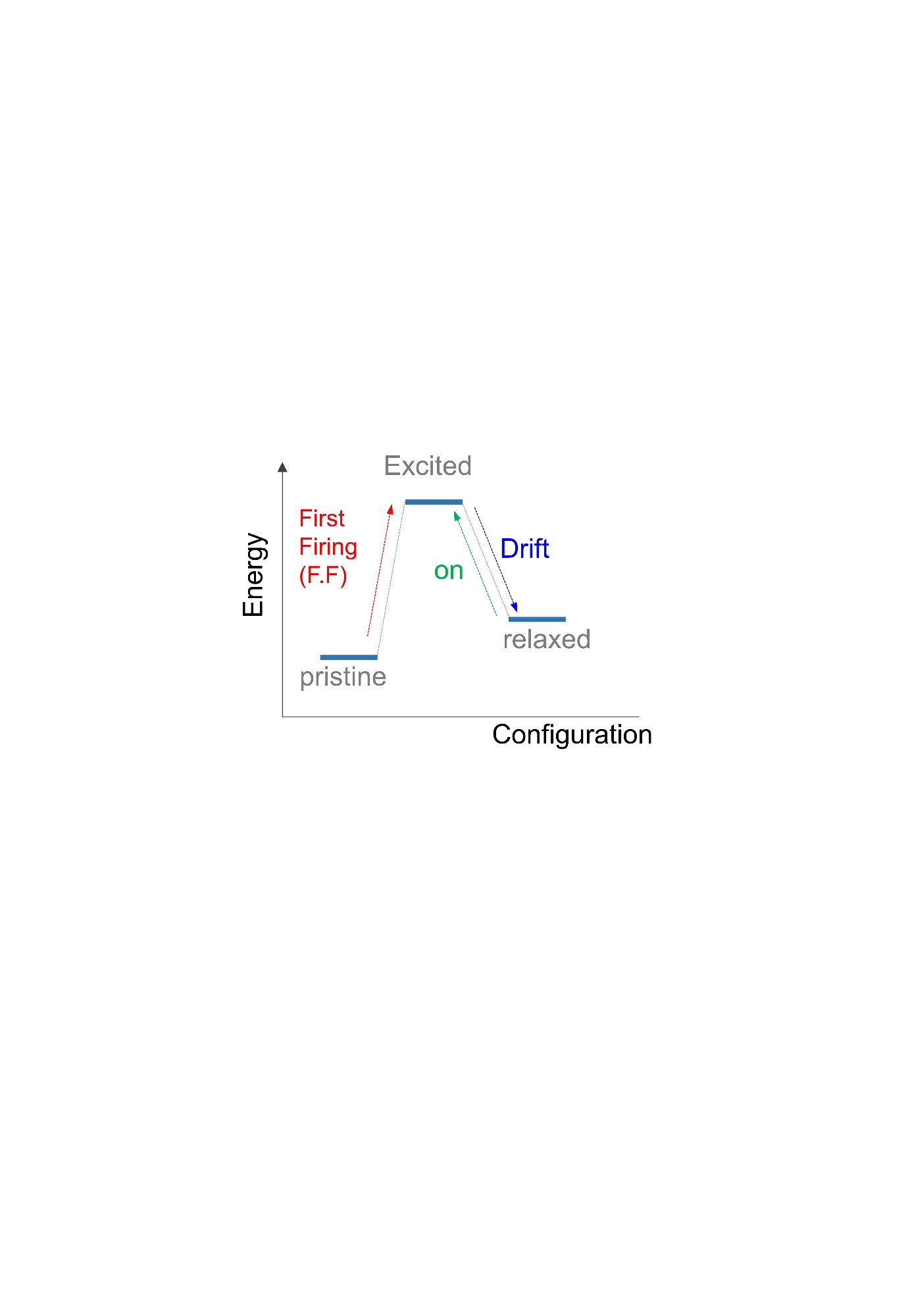


**Fig. S13.** Energy states of the investigated material. Illustration of the three energy states: pristine, excited, and relaxed states.

**Table S1.** Electrical properties of the Ge-As-Se-In-S-based selector devices.

| **Composition** | **Ge-As-Se** | **Ge-As-Se-In** | **Ge-As-Se-In-S** | **Ge-As-Se-In-S**  **(Optimized)** |
| --- | --- | --- | --- | --- |
| *I_off_* (nA) @ 0.6**V_th_* | 0.23 | 0.58 | 0.29 | 0.27 |
| *V_th_* drift (mV/dec) | 83.2 | 16.3 | 19.5 | 17.1 |
| Endurance (G) | 1 | >30 | >50 | >70 |

**Table S2.** For the alloying elements X in Ge-As-Se composition, we perform the DFT simulation to obtain the band gap (*E_g_*) and trap density (*N_T_*) parameters as averaged values of 10 different amorphous structure. The X composition is fixed to 5 % in Ge_20_As_30_Se_50_ compositions. Using the *E_g_* and *N_T_* as input parameters in TCAD simulation, we can calculate *V_th_* and *I_off_* of X-Ge-As-Se system. *ΔI_off_* (%) is determined as [*I_off (Ge-As-Se)_*- *I_off (X-Ge-As-Se)_*]/ *I_off (Ge-As-Se)_* × 100 (%), where *I_off (Ge-As-Se)_* and *I_off (X-Ge-As-Se)_* mean the leakage current of pure Ge-As-Se and X-Ge-As-Se, respectively.

| Elem. (*X*) | DFT | | TCAD | | ΔE_g_ (eV) | Δ*I_off_* (%) |
| --- | --- | --- | --- | --- | --- | --- |
|  | *E_g_* (eV) | *N_T_* (cm^-3^) | *V_th_* (V) | *I_off_* (nA) |  |  |
| Pure | 1.774 | 1.0.E+26 | 3.60 | 2.97E-10 | - | - |
| Li | 1.796 | 8.9.E+25 | 3.62 | 1.98E-10 | 0.022 | -33.4 |
| Be | 1.925 | 1.8.E+26 | 3.77 | 1.14E-10 | 0.151 | -61.7 |
| B | 1.860 | 2.3.E+26 | 3.68 | 4.68E-10 | 0.086 | 57.6 |
| C | 1.759 | 2.9.E+26 | 3.52 | 2.55E-09 | -0.015 | 760.2 |
| N | 1.707 | 9.3.E+25 | 3.52 | 4.81E-10 | -0.067 | 62.3 |
| O | 1.984 | 1.8.E+26 | 3.83 | 4.35E-11 | 0.210 | -85.3 |
| F | 1.809 | 2.1.E+26 | 3.61 | 7.43E-10 | 0.035 | 150.5 |
| Na | 1.898 | 2.4.E+26 | 3.73 | 2.83E-10 | 0.124 | -4.6 |
| Mg | 1.815 | 1.8.E+26 | 3.63 | 5.17E-10 | 0.041 | 74.4 |
| Al | 1.840 | 1.7.E+26 | 3.66 | 3.57E-10 | 0.066 | 20.5 |
| Si | 1.887 | 2.6.E+26 | 3.71 | 3.99E-10 | 0.113 | 34.7 |
| P | 1.830 | 1.7.E+26 | 3.65 | 4.17E-10 | 0.056 | 40.5 |
| S | 1.926 | 1.2.E+26 | 3.77 | 5.80E-11 | 0.152 | -80.4 |
| Cl | 1.853 | 1.4.E+26 | 3.68 | 2.16E-10 | 0.079 | -27.3 |
| K | 1.787 | 8.9.E+25 | 3.61 | 2.16E-10 | 0.013 | -27.2 |
| Ca | 1.808 | 1.2.E+26 | 3.63 | 3.23E-10 | 0.034 | 8.9 |
| Sc | 1.794 | 1.5.E+26 | 3.61 | 4.72E-10 | 0.020 | 59.1 |
| Ti | 1.575 | 2.3.E+26 | 3.29 | 6.66E-09 | -0.199 | 2146.5 |
| V | 1.387 | 3.5.E+26 | 2.96 | 3.10E-08 | -0.387 | 10356.5 |
| Cr | 1.348 | 6.8.E+26 | 2.74 | 1.25E-07 | -0.426 | 41911.8 |
| Mn | 1.485 | 8.9.E+26 | 3.23 | 4.53E-09 | -0.289 | 1426.3 |
| Fe | 1.556 | 6.0.E+26 | 3.07 | 4.50E-08 | -0.218 | 15086.1 |
| Co | 1.498 | 6.5.E+26 | 2.95 | 7.63E-08 | -0.276 | 25612.3 |
| Ni | 1.544 | 5.5.E+26 | 2.76 | 1.14E-08 | -0.230 | 3755.2 |
| Cu | 1.665 | 2.1.E+26 | 3.42 | 2.93E-09 | -0.109 | 888.9 |
| Zn | 1.802 | 3.0.E+26 | 3.59 | 1.63E-09 | 0.028 | 448.2 |
| Ga | 1.809 | 1.5.E+26 | 3.36 | 1.79E-10 | 0.035 | -39.8 |
| Ge | 1.752 | 1.2.E+26 | 3.57 | 4.95E-10 | -0.022 | 67.0 |
| As | 1.720 | 1.2.E+26 | 3.53 | 6.39E-10 | -0.054 | 115.3 |
| Se | 1.810 | 2.3.E+26 | 3.61 | 8.80E-10 | 0.036 | 196.5 |
| Br | 1.912 | 2.3.E+26 | 3.75 | 2.06E-10 | 0.138 | -30.4 |
| Rb | 1.763 | 1.2.E+26 | 3.58 | 4.32E-10 | -0.011 | 45.7 |
| Sr | 1.763 | 9.1.E+25 | 3.58 | 2.79E-10 | -0.011 | -5.9 |
| Y | 1.764 | 1.5.E+26 | 3.57 | 6.57E-10 | -0.010 | 121.3 |
| Zr | 1.581 | 1.5.E+26 | 3.34 | 2.70E-09 | -0.193 | 811.8 |
| Nb | 1.598 | 5.5.E+26 | 3.15 | 3.04E-08 | -0.176 | 10137.5 |
| Mo | 1.481 | 5.8.E+26 | 2.96 | 5.59E-08 | -0.293 | 18755.9 |
| Tc | 1.474 | 5.9.E+26 | 2.94 | 5.78E-08 | -0.300 | 19387.9 |
| Ru | 1.594 | 5.1.E+26 | 3.16 | 2.63E-08 | -0.180 | 8771.1 |
| Rh | 1.506 | 4.5.E+26 | 3.07 | 3.24E-08 | -0.268 | 10825.8 |
| Pd | 1.562 | 2.4.E+26 | 3.27 | 7.75E-09 | -0.212 | 2514.0 |
| Ag | 1.708 | 1.2.E+26 | 3.51 | 7.17E-10 | -0.066 | 141.6 |
| Cd | 1.834 | 2.1.E+26 | 3.65 | 5.84E-10 | 0.060 | 96.9 |
| In | 1.753 | 1.8.E+26 | 3.55 | 9.90E-10 | -0.021 | 233.6 |
| Sn | 1.730 | 1.7.E+26 | 3.52 | 1.20E-09 | -0.044 | 305.9 |
| Sb | 1.771 | 1.7.E+26 | 3.58 | 1.59E-09 | -0.003 | 435.2 |
| Te | 1.768 | 2.0.E+26 | 3.56 | 1.12E-09 | -0.006 | 275.9 |
| I | 1.768 | 8.5.E+25 | 3.59 | 2.41E-10 | -0.006 | -18.6 |
| Ba | 1.776 | 2.1.E+26 | 3.57 | 1.11E-09 | 0.002 | 273.7 |
| La | 1.703 | 1.2.E+26 | 3.50 | 7.90E-10 | -0.071 | 166.2 |
| Hf | 1.733 | 1.5.E+26 | 3.53 | 8.33E-10 | -0.041 | 180.9 |
| Ta | 1.605 | 1.4.E+26 | 3.37 | 2.18E-09 | -0.169 | 636.2 |
| W | 1.418 | 5.0.E+26 | 2.92 | 5.19E-08 | -0.356 | 17412.7 |
| Re | 1.605 | 7.8.E+26 | 3.06 | 5.30E-08 | -0.169 | 17765.4 |
| Os | 1.545 | 7.1.E+26 | 2.99 | 5.98E-08 | -0.229 | 20048.7 |
| Ir | 1.679 | 6.9.E+26 | 3.23 | 2.59E-08 | -0.095 | 8618.0 |
| Pt | 1.727 | 5.1.E+26 | 3.39 | 9.92E-09 | -0.047 | 3243.9 |
| Au | 1.728 | 1.7.E+26 | 3.52 | 1.24E-09 | -0.046 | 319.7 |
| Hg | 1.786 | 2.0.E+26 | 3.59 | 9.18E-10 | 0.012 | 209.4 |
| Tl | 1.843 | 1.4.E+26 | 3.67 | 2.54E-10 | 0.069 | -14.4 |
| Pb | 1.811 | 1.8.E+26 | 3.63 | 5.50E-10 | 0.037 | 85.4 |
| Bi | 1.710 | 2.0.E+26 | 3.48 | 1.92E-09 | -0.064 | 547.2 |
| Po | 1.681 | 2.0.E+26 | 3.45 | 2.47E-09 | -0.093 | 732.9 |
| At | 1.733 | 1.4.E+26 | 3.54 | 7.90E-10 | -0.041 | 166.4 |
